# Genetic subtraction reveals divergent pathways and targets in anxiety-related and anxiety-independent TMD

**DOI:** 10.1186/s10194-026-02304-3

**Published:** 2026-03-09

**Authors:** Yu Cao, Xin Yang, Peter Svensson, Raymond Wong Chung Wen, Timothy Jie Han Sng, Intekhab Islam, Wei Han, Xingmei Feng, Bozhi Hou, Yuehua Li, Lei Zheng

**Affiliations:** 1 Faculty of Dentistry, National University of Singapore, Singapore, 119085 Singapore; 2 Department of Oral and Maxillofacial Surgery, Nanjing Stomatological Hospital, Affiliated Hospital of Medical School, Nanjing University, 30 Zhongyang Road, Nanjing, 210008 China; 3 Department of Stomatology, Medical School of Nantong University, Affiliated Hospital of Nantong University, Nantong, 226001 China

**Keywords:** Temporomandibular disorders, Anxiety, Chronic pain symptom, GWAS-by-subtraction, Neurogenetic pathways, Multi-omics integration

## Abstract

**Background:**

Temporomandibular disorders (TMD) show substantial clinical and genetic overlap with anxiety, yet it remains unclear whether TMD risk reflects shared anxiety-related liability or distinct anxiety-independent genetic mechanisms. Disentangling these components is essential for understanding TMD heterogeneity beyond symptom-based classifications.

**Methods:**

We applied GWAS-by-subtraction using genome-wide summary statistics for TMD (20,799 cases and 479,549 controls; FinnGen Release 12) and anxiety disorders (74,973 cases and 400,243 controls), partitioning TMD heritability into two orthogonal latent components: an anxiety-dependent factor (F_Anxiety_) and an anxiety-independent factor (F_Non-Anxiety_). To delineate the mechanisms underlying each component, we integrated fine-mapping, transcriptome- and proteome-wide association analyses, genetic colocalization, brain imaging–genetics, and single-cell RNA sequencing from human embryonic temporomandibular joint tissue.

**Results:**

Anxiety showed significant genetic correlation with TMD (rg = 0.4417, *p* = 1.98 × 10^− 1 9^) and accounted for 19.50% of TMD heritable variance. F_Anxiety_ yielded multiple genome-wide significant loci (*CNTNAP5*, *PCLO*, *PRSS16*, *BTN1A1*, *RAB27B*), whereas F_Non-Anxiety_ produced a single independent signal near *GPNMB*, demonstrating sharply divergent genetic architectures. Multi-omic integration identified *RAB27B* as a driver of the anxiety-related pathway, implicating synaptic vesicle trafficking and neuroimmune regulation, while *GPNMB* and *KLHL7* supported anxiety-independent pathways involving musculoskeletal remodeling and peripheral inflammation. BrainXcan analyses showed that F_Anxiety_ predominantly affected limbic and external capsule microstructure, whereas F_Non-Anxiety_ mapped to thalamic–sensorimotor white matter networks. Single-cell mapping further revealed distinct enrichment patterns of *RAB27B*, *KLHL7*, and *GPNMB* across TMJ cell types.

**Conclusion:**

These findings demonstrate that TMD genetic liability comprises separable anxiety-related and anxiety-independent dimensions with distinct molecular, neurostructural, and cellular signatures. Rather than defining clinical subtypes, these latent components represent associative dimensions of genetic risk at the population level. This integrative framework clarifies the genetic architecture underlying TMD heterogeneity and provides a foundation for future studies integrating individual-level phenotyping to assess clinical relevance and causal mechanisms.

**Graphical Abstract:**

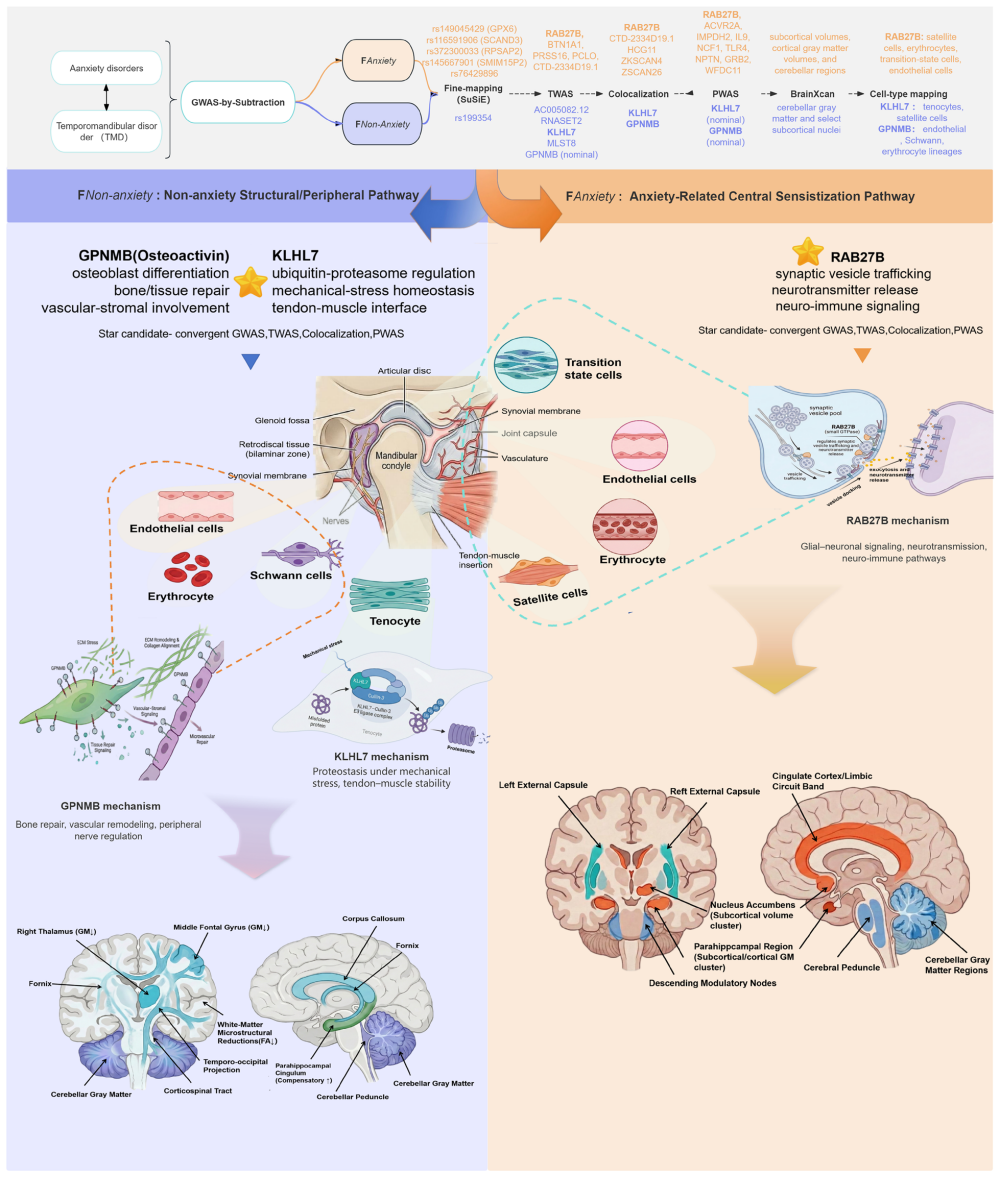

**Supplementary Information:**

The online version contains supplementary material available at 10.1186/s10194-026-02304-3.

## Background

Temporomandibular disorders (TMDs) represent a group of clinically diverse and orofacial pain conditions involving the temporomandibular joint, masticatory muscles, and associated tissues. Globally, TMD affects up to one-third of adults and imposes a substantial burden through chronic pain, functional limitation, and impaired quality of life [1, 2]. Although the classical understanding of TMD pain has focused on peripheral biomechanical and inflammatory contributors, increasing evidence highlights the importance of central pain modulation and psychosocial factors in shaping symptom severity and chronicity [3–6]. These findings underscore the heterogeneity of TMD and the need to better understand the diverse mechanisms driving its clinical presentation [7, 8]. This is indeed in line with a recent proposal that TMD pain may represent a spectrum of mechanistically different pain conditions, spanning from nociceptive types of pain based on peripheral nociceptive inputs to oncoplastic pain with a greater importance of central sensitization phenomena and imbalanced descending inhibitory and facilitatory drive [9].

TMD pain has been suggested for more than three decades to be best understood in a biopsychosocial context and assessed on a physical Axis I and a psychosocial distress Axis II [10–12]. Among psychosocial influences, anxiety is one of the most consistently reported accompanying features in TMD [13, 14]. Higher anxiety levels are associated with elevated pain sensitivity, widespread pain, heightened somatosensory amplification, and poorer clinical outcomes [15–18]. However, despite its high prevalence, not all individuals with TMD exhibit anxiety. A considerable proportion of TMD patients report minimal or no anxiety symptoms, even in the presence of comparable orofacial pain [19]. This clinically recognized divergence raises a fundamental question: does the presence of anxiety correspond to a biologically distinct form of TMD or is anxiety merely a co-occurring trait that modulates symptom perception without conferring unique genetic contributions. Despite decades of research on TMD pain pathophysiology, this question remains entirely unanswered. Existing genome-wide association studies (GWAS) treat TMD pain as a unitary phenotype and therefore cannot differentiate genetic risk shared by all TMD patients from genetic factors specifically associated with anxiety in TMD [20–23]. As a result, the field lacks a clear understanding of whether anxiety-related variation represents an intrinsic genetic component of TMD pain, or whether TMD pain with and without anxiety share an identical genetic architecture. Resolving this gap is crucial for clarifying biological heterogeneity within TMD pain, identifying distinct mechanistic pathways, and informing more targeted diagnostic and therapeutic approaches.

In this study, we address this critical knowledge gap by applying GWAS-by-Subtraction, a statistical framework that decomposes overlapping genetic signals into distinct latent components [24]. Leveraging large-scale genetic data, we isolate the portion of TMD genetic liability that is specifically related to anxiety from the portion that is independent of anxiety. This approach enables us to determine whether TMD with anxiety carries a unique genetic signature and to identify biological pathways that differentiate anxiety-associated and anxiety-independent TMD. By resolving the genetic architecture underlying these two clinically meaningful manifestations of TMD, our work provides new insight into the mechanisms driving TMD pain heterogeneity and establishes a foundation for biologically informed precision management of TMD.

## Materials and methods

### Data source

GWAS summary statistics for temporomandibular joint disorders (TMD) were obtained from the FinnGen study (Release 12; https://www.finngen.fi/en). FinnGen is a large-scale public–private initiative that integrates genomic data from more than 500,000 Finnish biobank participants with nationwide health registry information to investigate disease mechanisms and genetic susceptibility [25]. In FinnGen, TMD was defined using the International Classification of Diseases, 10th revision (ICD-10) code K07.6, a broad diagnostic category that encompasses several temporomandibular joint conditions, including TMJ derangement, TMJ painful and non-painful diagnoses, which captures the full clinical spectrum of TMD.The data set comprised 500,348 individuals, including 20,799 cases and 479,549 controls.

For anxiety, GWAS summary statistics were obtained from a meta-analysis of five European-ancestry cohorts [26]. Case definitions varied across cohorts, encompassing ICD-10 or DSM diagnoses, lifetime or self-reported anxiety disorders, and proxy phenotypes based on screening tools such as the GAD-2. Full details of case definitions are provided in the original publication [26]. In total, the meta-analysis included 74,973 cases (28,392 proxy cases) and 400,243 controls (146,771 proxy controls).

To reduce potential confounding from population stratification, only individuals of European ancestry were included. All summary statistics were harmonized to dbSNP build 157 using GWASLab tool [27] to ensure consistency of SNP identifiers, alleles, and genomic coordinates across data sets.

### GWAS-by-subtraction

Starting from GWAS summary statistics for TMD and anxiety disorders, the method “subtracts” the influence of anxiety from each SNP’s effect on TMD to estimate SNP associations with TMD independent of anxiety. This framework generates an alternative set of summary statistics that approximate those expected from a GWAS of TMD conditioned on anxiety. The theoretical basis of GWAS-by-subtraction has been described previously [28].

We implemented an adapted version of GWAS-by-subtraction using **GSUB**, a command-line tool that applies closed-form solutions to decompose SNP effects into orthogonal latent genetic components derived from two traits [29]. GWAS summary statistics for TMD were obtained from the FinnGen study, while summary statistics for anxiety disorders were drawn from a large-scale meta-analysis. In this model, the GWAS summary statistics for both traits were jointly regressed onto two latent factors: an anxiety-related genetic component (F_Anxiety_) and a residual, anxiety-independent genetic component specific to TMD (F_Non-Anxiety_).

Factor loadings of TMD and anxiety onto each latent component were explicitly estimated at the model level, following standard structural equation modeling principles applied to the genetic correlation matrix. To ensure interpretability of the decomposition, the genetic covariance between F_Anxiety_ and F_Non-Anxiety_ was fixed at zero, enforcing orthogonality between the two latent factors. This orthogonality constraint is a defining feature of the GWAS-by-subtraction framework and ensures that the anxiety-independent component captures only genetic variation in TMD that is uncorrelated with anxiety, rather than implying biological or clinical independence between underlying mechanisms.

Under this two-trait orthogonal model specification, the proportion of TMD heritable variance attributable to anxiety-related genetic liability was quantified as the square of the standardized loading of TMD on F_Anxiety_. This quantity is mathematically equivalent to the squared genetic correlation (rg^2^) between TMD and anxiety, because all shared genetic variance between the two traits is fully captured by F_Anxiety_.

**Fig. 1:**
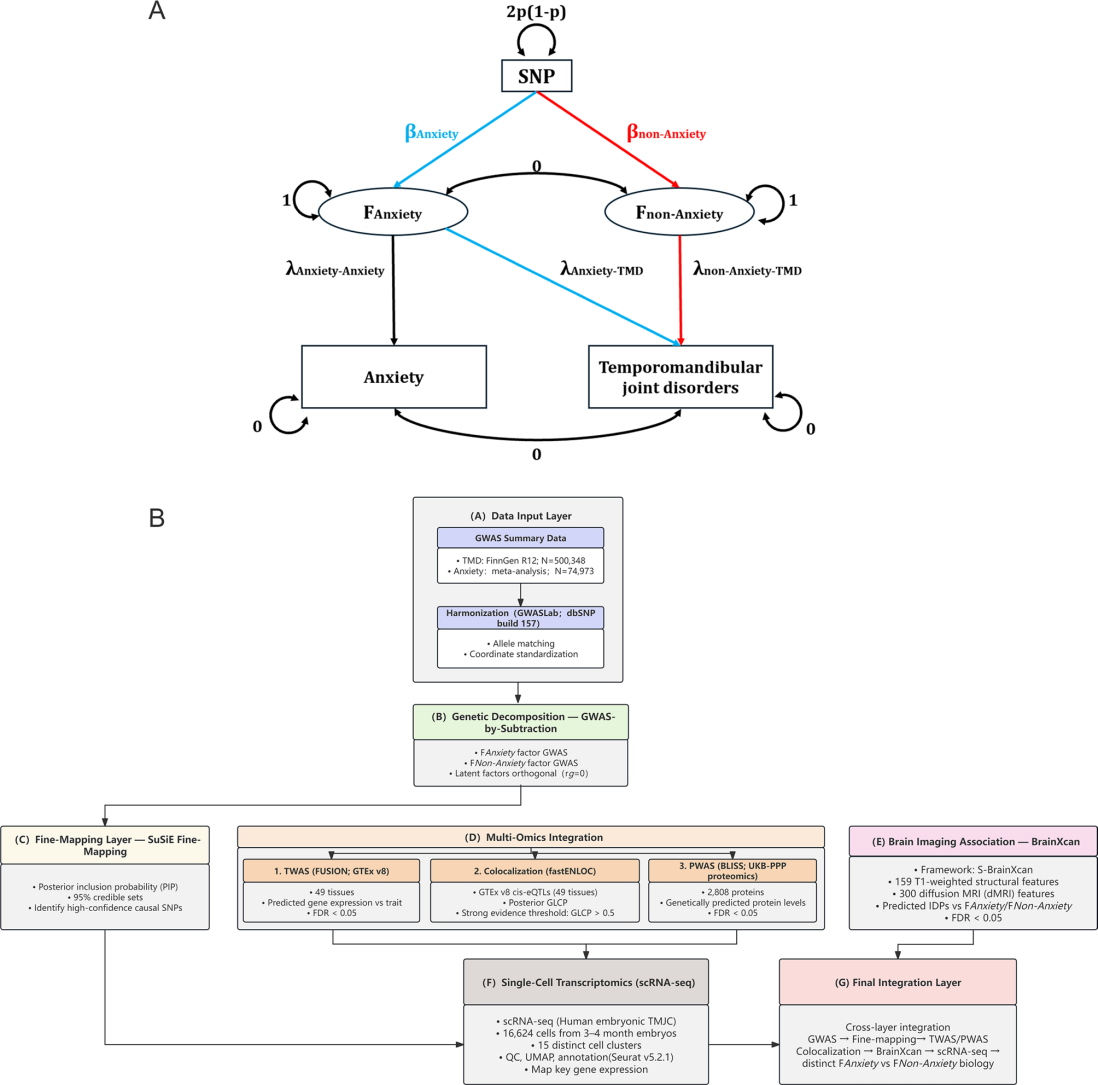
Study design and analytical framework for dissecting anxiety-related and anxiety-independent genetic components of temporomandibular disorders. (**A**) Conceptual framework of GWAS-by-subtraction applied to temporomandibular disorders (TMD) and anxiety. Structural equation model illustrating the decomposition of genetic effects on TMD into anxiety-related (FAnxiety) and anxiety-independent (F_Non-Anxiety_) components. SNP variance is modeled as 2p(1–p), where p is the reference allele frequency. SNP effects on the two latent factors are denoted as βAnxiety (blue) and βnon-anxiety (red). F_Anxiety_ captures the genetic variance in TMD mediated through anxiety, while F_Non-Anxiety_ represents the residual genetic variance in TMD independent of anxiety. Path loadings (λ) indicate factor–phenotype relationships: λAnxiety–anxiety and λAnxiety–TMD link F_Anxiety_ to observed anxiety and TMD, respectively, while λnon-Anxiety–TMD links F_Non-Anxiety_ to TMD. By construction, F_Anxiety_ and F_Non-Anxiety_ are orthogonal (rg = 0), ensuring independence between the shared and residual genetic components. (**B**) Multi-omics integrative analysis workflow for anxiety-related and anxiety-independent TMD

Each latent factor was subsequently regressed on SNPs genome-wide, yielding two sets of factor-specific GWAS summary statistics. SNPs with *p* < 5 × 10^− 8^ were considered genome-wide significant. Independent lead SNPs were identified by LD clumping using an r^2^ threshold of < 0.01 within a ± 1 Mb window. The overall structure of the GWAS-by-subtraction model, including factor loadings, SNP pathways, and orthogonality constraints, is illustrated in Fig. 1 A

Importantly, the anxiety-related and anxiety-independent components represent a statistical decomposition of genetic liability at the population level rather than empirically defined or directly observable clinical subgroups. These latent factors describe distinct dimensions of genetic risk and do not imply that individual patients can be categorically classified into anxiety-related or anxiety-independent TMD subtypes. Translation of these genetic components into clinically actionable subgroups will require future studies integrating individual-level genetic data with more granular phenotypic and clinical information.

### Fine-mapping of latent factors

To refine loci identified from the factor GWAS of F_Anxiety_ and F_Non-Anxiety_, we applied Bayesian fine-mapping using the Sum of Single Effects (SuSiE) model to achieve single-variant resolution [30]. Fine-mapping assigns posterior inclusion probabilities (PIPs) to individual SNPs, allowing distinction between variants most likely to be causal and those correlated through linkage disequilibrium (LD).

The SuSiE model decomposes regional association signals into multiple “single-effect” components, while explicitly accounting for local LD structure, and generates 95% credible sets of candidate causal variants. Single-variant resolution was defined as either (i) one SNP achieving PIP > 0.8 or (ii) a 95% credible set containing only a single variant.

Fine-mapping was performed for all lead loci identified from the GWAS of F_Anxiety_ and F_Non-Anxiety_. For each lead SNP, we extracted genomic windows of ± 1 Mb from harmonized summary statistics, and estimated LD patterns using the European reference panel from the 1000 Genomes Project (Phase 3).

### Transcriptome-wide association study (TWAS) of latent factors

To further prioritize putative genes underlying the genetic signals identified in the factor GWAS of F_Anxiety_ and F_Non-Anxiety_, we performed transcriptome-wide association studies (TWAS) using the FUSION framework [31]. TWAS integrates GWAS summary statistics with gene expression reference weights to test whether predicted gene expression levels are associated with the trait of interest.

We used the FUSION software with precomputed expression weights from GTEx v8 [32], spanning 49 tissues. For each gene, FUSION predicts expression levels as a linear function of cis-SNPs within ±500 kb of the transcription start and end sites, using elastic net, LASSO, and BLUP models trained on GTEx reference data. Association statistics were computed by integrating SNP-level GWAS z-scores from the F_Anxiety_ and F_Non-Anxiety_ factor GWAS with SNP-expression weight matrices, while accounting for linkage disequilibrium (LD) using the European 1000 Genomes reference panel. TWAS was conducted separately for F_Anxiety_ and F_Non-Anxiety_. Genes with a within-tissue false discovery rate (FDR) < 0.05 were considered significant.

### Colocalization analysis

The objective of the colocalization analysis was to evaluate whether genetic associations for F_Anxiety_ and F_Non-Anxiety_ shared the same underlying causal variants with cis-eQTLs, thereby implicating gene regulation as a mediator of these genetic effects. We applied the Bayesian hierarchical framework implemented in **fastENLOC** [33], which integrates GWAS and QTL summary statistics to jointly estimate enrichment, fine-mapping, and colocalization probabilities.

Credible sets derived from SuSiE fine-mapping were used as GWAS input, while cis-eQTL data were obtained from GTEx v8 across 49 tissues. Only variants present in both the factor GWAS and eQTL data sets were analyzed. The analysis proceeded in three steps: (i) enrichment estimation of eQTL annotations among GWAS signals, (ii) Bayesian fine-mapping within LD blocks to assign PIPs to candidate variants, and (iii) computation of gene-locus colocalization probabilities (GLCPs), which quantify the probability that the same causal variant drives both the GWAS and eQTL associations.

A GLCP > 0.5 was predefined as the primary threshold indicating strong evidence of colocalization and was used for confirmatory interpretation across all analyzes. Results exceeding this threshold were considered robust evidence of shared causal variants between genetic associations and gene expression. Given the relatively lower statistical power of the F_Non-Anxiety_ component, we additionally conducted exploratory colocalization analyzes using a relaxed threshold (GLCP > 0.2).

### Proteome-wide association study (PWAS)

To extend SNP- and gene-level findings to the protein layer, we conducted proteome-wide association studies (PWAS) using the BLISS framework (Biomarker expression Level Imputation using Summary statistics) [34]. BLISS imputes genetically predicted plasma protein levels from cis-pQTL summary statistics, enabling systematic testing of protein–trait associations without individual-level proteomic data.

We applied European-ancestry BLISS pretrained models derived from large-scale proteomic reference cohorts, including the UK Biobank Plasma Proteome Project (UKB-PPP), comprising 49,341 individuals and 2808 plasma proteins. These predictive models were trained in the original BLISS framework using cis-acting SNPs within ±1 Mb of each gene, retaining only proteins with significant cis-heritability and adequate predictive performance.

Genetically predicted protein levels from each reference model were then integrated separately with GWAS summary statistics from the F_Anxiety_ and F_Non-Anxiety_ factor analyzes. Association statistics were computed using burden-type Z tests, as implemented in BLISS, with multiple-testing correction applied across the proteome. Proteins surpassing the significance threshold (FDR < 0.05) were considered candidate mediators of the anxiety-related (F_Anxiety_) or anxiety-independent (F_Non-Anxiety_) genetic components of TMD. Proteins supported by convergent evidence across PWAS, TWAS, and colocalization analyzes were further prioritized as high-confidence candidates for functional interpretation and downstream follow-up.

### Brain imaging feature association via BrainXcan

To investigate whether the anxiety-related (F_Anxiety_) and anxiety-independent (F_Non-Anxiety_) genetic components of TMD are linked to structural and microstructural features of the brain, we applied BrainXcan [35]. BrainXcan leverages GWAS summary statistics in combination with genetically trained prediction models of MRI-derived brain imaging phenotypes (IDPs) to test trait–brain feature associations.

Pretrained genetic predictors were obtained from the UK Biobank imaging cohort (*N* = 24,409 European individuals), which included 159 T1-weighted structural features (cortical, subcortical, cerebellar, and global volumes) and 300 diffusion MRI-derived features (neurite density, anisotropy, dispersion, and connectivity). For each modality, principal components were used to capture brain-wide features, while residuals represented region-specific effects. Prediction models were trained using ridge regression on HapMap3 SNPs (MAF > 0.01) and retained features with cross-validated prediction performance (correlation > 0.1).

GWAS z-scores from the FAnxiety and FNon-Anxiety factor analyses were integrated with brain feature prediction weights, with linkage disequilibrium estimated from the same UK Biobank imaging reference panel. Association statistics were computed for each IDP, yielding z-scores, standard errors, and *p*-values for the genetically predicted feature–trait relationships.

Significant associations (FDR < 0.05 across features) were interpreted as evidence that genetic liability underlying FAnxiety or FNon-Anxiety is linked to specific brain structural or microstructural characteristics.

### Single-cell–Informed dissection of TMD molecular pathways

To further elucidate the putative cellular and molecular mechanisms underlying TMD, we incorporated single-cell RNA sequencing (scRNA-seq) data from a publicly available data set of human embryonic temporomandibular joint condyle (TMJC) tissue [36]. At present, publicly available scRNA-seq data sets for adult human temporomandibular joint, jaw muscle, or tendon tissues are not yet available; therefore, our single-cell analysis is restricted to TMJ-derived embryonic cell populations, which are interpreted as reflecting developmental programming and early tissue biology rather than adult tissue states.

This data set comprises 16,624 cells from 3- and 4-month-old human embryonic TMJC and delineates 15 distinct cell clusters. Preprocessing, quality control, normalization, and cell-type annotation were performed following the original published protocol, and we directly utilized the author-curated, quality-controlled expression matrix and cluster annotations. In the original study, low-quality cells were filtered using standard criteria (nFeature_RNA > 200 and < 4000; percent.mt < 50; nCount_RNA < 20,000), expression data were normalized using SCTransform, and highly variable genes were selected for downstream analyzes. Cell-type identities were assigned based on established marker genes reported in the original publication. Uniform Manifold Approximation and Projection (UMAP) was applied for dimensionality reduction and visualization.

To integrate the single-cell information with the GWAS-by-subtraction results, we examined the expression patterns of prioritized candidate genes across annotated cell types using feature plots and violin plots. All analyzes were performed using the Seurat R package (v5.2.1) [37].

A schematic overview of the complete analytical workflow is shown in Fig. 1B.

## Result

### Decomposition of TMD genetic liability into anxiety-dependent and anxiety-independent components

Before identifying component-specific loci, we first evaluated the overall genetic relationship between anxiety and temporomandibular disorders (TMD). The two traits showed a substantial genetic correlation (rg = 0.4417, *p* = 1.98 × 10^−19^). Within the GWAS-by-subtraction framework, the anxiety-related component (F_Anxiety_) accounted for 19.50% of the heritable variation in TMD (factor loading = 0.4417), whereas the anxiety-independent component (F_Non-Anxiety_) explained the remaining 80.50% (factor loading = 0.8972).

Building on this shared genetic architecture, we then used GWAS-by-subtraction to separate TMD into an anxiety-dependent (F_Anxiety_) and an anxiety-independent (F_Non-Anxiety_) component and began examining their genome-wide association results.

**Fig. 2:**
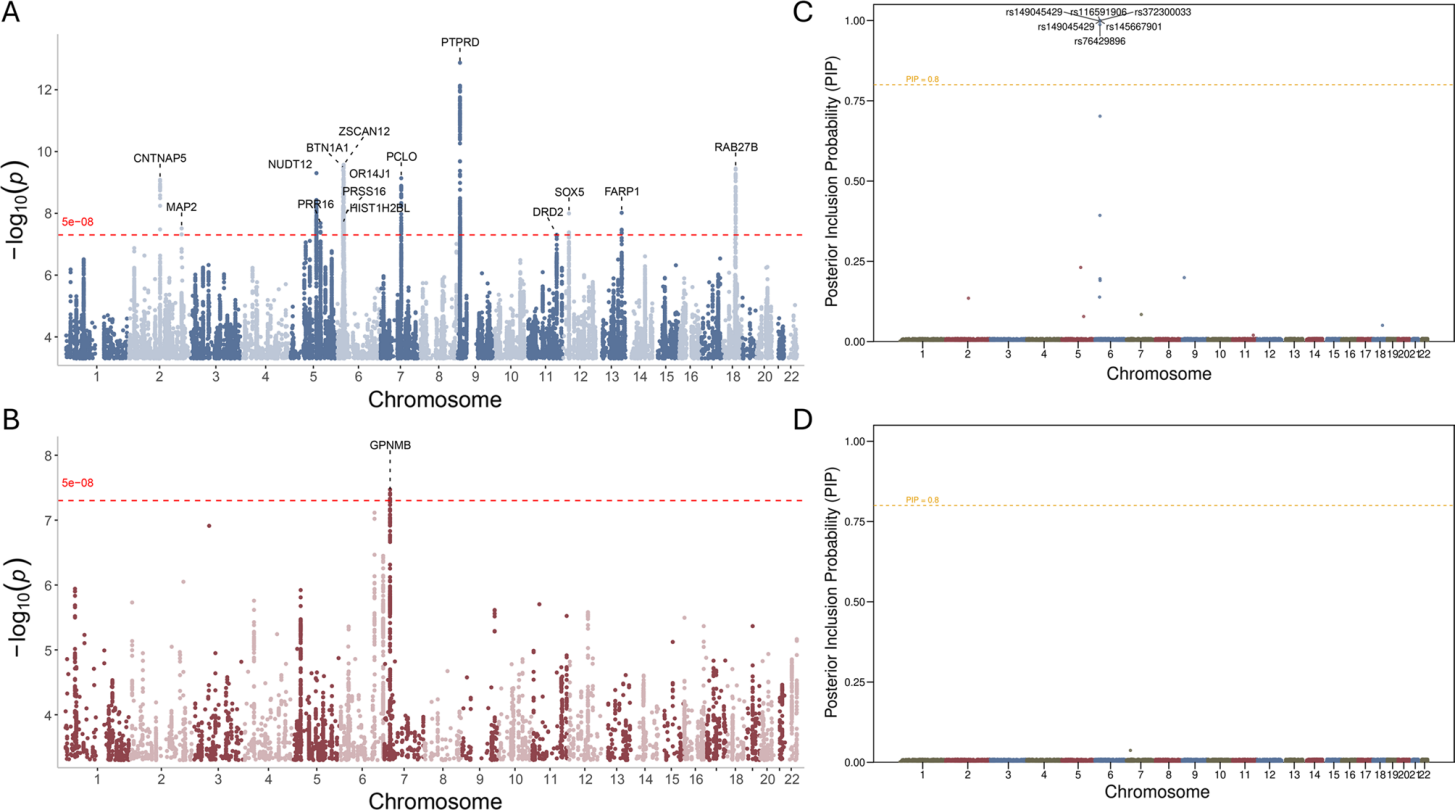
Integrated genome-wide association and SuSiE fine-mapping of anxiety-dependent and anxiety-independent genetic components of TMD. (**A**) Manhattan plot for the anxiety-dependent (F_Anxiety_ pathway) component. The red dashed line denotes the genome-wide significance threshold (*p* = 5 × 10^8^). (**B**) Manhattan plot for the anxiety-independent (F_Non-Anxiety_ pathway) component, following the same conventions. (**C**) in the F_Anxiety_ pathway, multiple loci exceed the confidence threshold (PIP > 0.8), with significant signals annotated, indicating focused genetic architecture. (**D**): In the F_Non-Anxiety_ pathway, no loci surpass the PIP threshold, suggesting weaker genetic signals or a polygenic distribution pattern

We applied the standard genome-wide significance threshold (*p* < 5 × 10^−8^) to identify significant loci across the genome. In the F_Anxiety_ pathway (Fig. 2A, Supplementary Table S1), 15 SNPs reached genome-wide significance. The candidate genes located near these significant loci included *CNTNAP5, MAP2, PRR16, NUDT12, ZSCAN12, BTN1A1, OR14J1, PRSS16, HIST1H2BL, PCLO, PTPRD, DRD2, SOX5, FARP1, and RAB27B.*

In contrast, in the F_Non-Anxiety_ pathway (Fig. 2B, Supplementary Table S1), we identified one independent genome-wide significant SNP, whose nearest gene was *GPNMB.*

### Fine-mapping reveals distinct causal architectures for anxiety-dependent and anxiety-independent TMD

To further refine these genome-wide signals and identify putative causal variants underlying the F_Anxiety_ and F_Non-Anxiety_ components, we performed Bayesian fine-mapping using the SuSiE framework. For the F_Anxiety_ component, 20 high-confidence causal candidates were identified (Fig.2 C, Supplementary Table S2), including rs149045429, rs116591906, rs372300033, rs145667901, and rs76429896, located near the *GPX6, SCAND3, RPSAP2,* and *SMIM15P2* genes, respectively. Notably, rs149045429, located on chromosome 6 (position: 28,490,915), exhibited an exceptionally high PIP value (0.998) in the SuSiE analysis, strongly supporting its causal role in the anxiety-related TMD pathway.

In contrast, the F_Non-Anxiety_ analysis (Fig. 2D, Supplementary Table S2) did not reveal any variant with PIP > 0.8; however, one 95% credible set containing a single SNP (rs199354) was identified, suggesting a more limited or distinct causal genetic basis for the anxiety-independent component.

### TWAS highlights component-specific regulatory gene signatures

To translate variant-level associations into gene-expression–level insights and identify transcriptionally mediated effects for each latent component, we conducted TWAS using the FUSION framework.

**Fig. 3:**
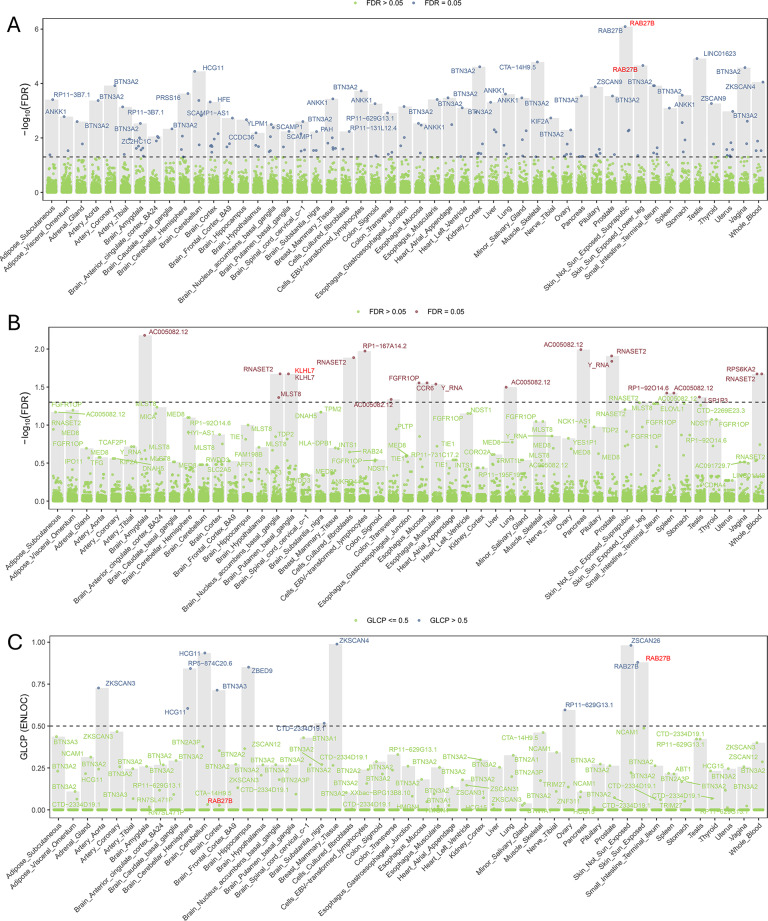

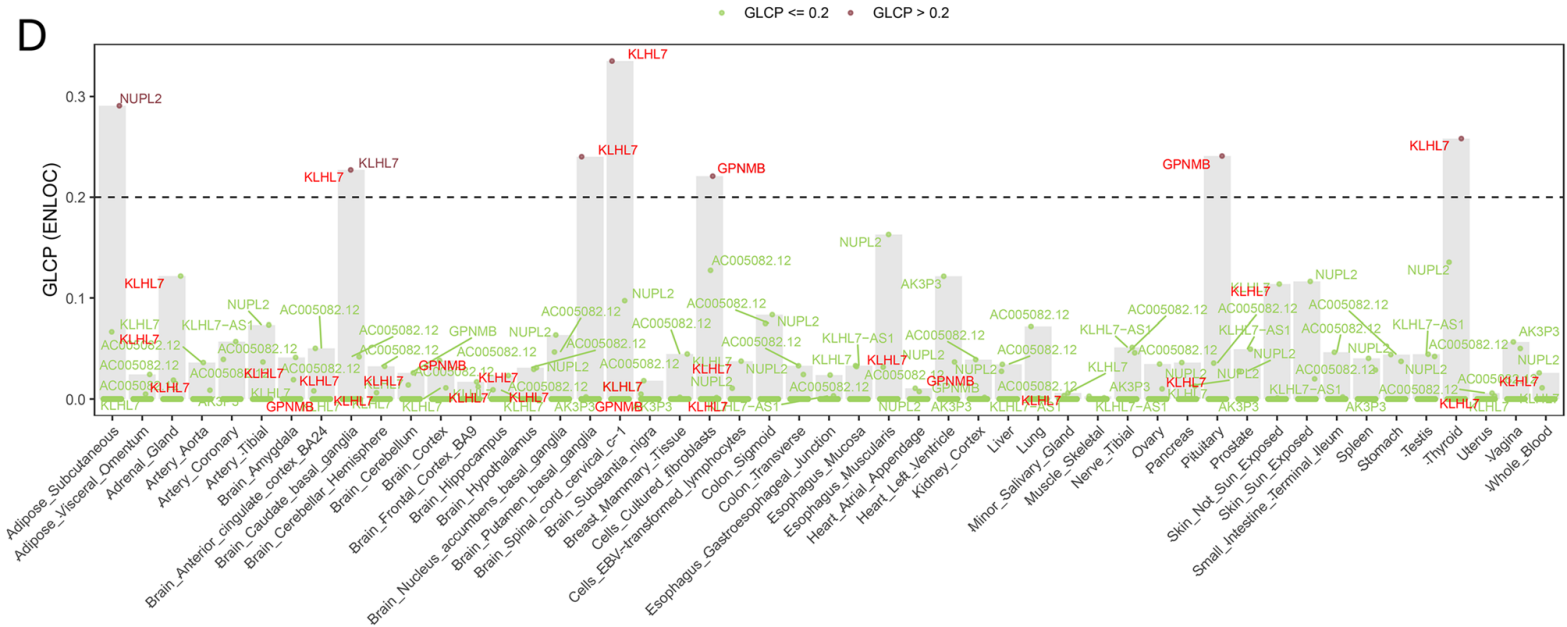
Integrated TWAS and fastENLOC colocalization analysis across 49 GTEx v8 tissues. (**A**): Genes significantly associated with the F_Anxiety_ pathway (FDR < 0.05), including *RAB27B*, *PCLO*, CTD-2334D19.1, *BTN1A1*, and *PRSS16*. (**B**): Genes significantly associated with the F_Non-Anxiety_ TMD pathway (FDR < 0.05); *GPNMB* shows nominal associations (*p* < 0.05) across 25 tissues. (**C**) Colocalization results for F_Anxiety_. Blue dots indicate high-confidence colocalized genes (gene-level colocalization probability, GLCP > 0.5), while green dots represent genes with lower confidence (GLCP ≤ 0.5). The dashed line marks the high-confidence threshold (GLCP = 0.5). TWAS-significant genes, including *HCG11*, ZKSCAN4, BTN3A3, RP5-874C20.6, *RAB27B*, ZSCAN26, ZKSCAN3, RP11-629G13.1, CTD-2334D19.1, are labeled. (**D**), Colocalization results for F_Non-Anxiety_. Red dots represent genes showing suggestive colocalization (GLCP > 0.2), and green dots indicate genes with lower probability (GLCP ≤ 0.2). The dashed line denotes the visualization threshold (GLCP = 0.2). Genes such as *KLHL7* and *GPNMB* showing potential colocalization in brain tissues are highlighted

We performed cross-tissue analyses to prioritize genes associated with the F_Anxiety_ component. After FDR correction (FDR < 0.05), we identified 1355 FDR-significant gene–tissue associations (420 unique genes) across multiple tissues (Fig. 3A; Supplementary Table S3). Several genes showed significant associations across both brain and peripheral tissues. *PRSS16* was negatively associated with the F_Anxiety_ component across multiple tissues, including the cerebellum hemisphere (Z = −5.39, FDR = 0.00023), cerebellum (Z = −5.24, FDR = 0.00042), and prostate (Z = −5.23, FDR = 0.00028). In testis tissue, *CTD-2334D19.1* was positively associated (Z = 4.87, FDR = 0.0023), whereas *BTN1A1* showed a negative association (Z = −4.51, FDR = 0.00796). *PCLO* exhibited tissue-dependent directions of association, showing negative associations in basal ganglia nuclei (nucleus accumbens and caudate; Z = −4.88, FDR = 0.0032) and positive associations in the stomach (Z = 4.83, FDR = 0.0027). *BTN3A2* also demonstrated significant associations across multiple tissues.

Notably, four genes—*RAB27B, BTN1A1, PRSS16, and PCLO*—overlapped with loci identified in the GWAS-by-subtraction analysis, providing convergent evidence and strengthening confidence in these associations.

In the F_Non-Anxiety_ pathway (Fig. 3B, Supplementary Table S3), 23 gene–tissue associations (13 unique genes) reached FDR-significance. Among them, *AC005082.12* showed significant negative associations across multiple tissues, including the amygdala (Z = −4.72, FDR = 0.0066). In addition, *RNASET2*, *KLHL7*, and *MLST8* exhibited significant negative associations in brain regions such as the nucleus accumbens and putamen of the basal ganglia.

Notably, *GPNMB*, which was annotated at the single genome-wide significant locus for the F_Non-Anxiety_ component, showed nominal-significant associations (*p* < 0.05) across 25 tissues but did not remain significant after FDR correction (FDR > 0.05).

### Colocalization analysis identifies shared regulatory signals across genetic components

To determine whether these TWAS-implicated expression signals share the same underlying causal variants as the genetic associations, we next conducted colocalization analysis across GTEx v8 tissues.

In the F_Anxiety_ pathway, strong colocalization was observed for several TWAS-significant genes, including *HCG11* (Brain_Cerebellum, GLCP = 0.94), *ZKSCAN4* (Breast_Mammary_Tissue, GLCP = 0.99), and *ZSCAN26* (Skin_Not_Sun_Exposed, GLCP = 0.98), indicating shared causal variants influencing gene expression in these tissues (Fig. 3C, Supplementary Table S4). Notably, *RAB27B* and *CTD-2334D19.1*, which were identified in both GWAS and TWAS analyzes, were further validated through colocalization, providing convergent evidence for a regulatory mechanism underlying the anxiety-related genetic component of TMD.

In contrast, no gene–tissue pair in the F_Non-Anxiety_ component exceeded the predefined GLCP > 0.5 threshold Given the relatively lower statistical power of the F_Non-Anxiety_ component, we additionally present exploratory colocalization results using a relaxed threshold (GLCP > 0.2) (Fig. 3D, Supplementary Table S4). Under this exploratory criterion, *KLHL7* (Brain_Caudate_basal_ganglia, GLCP = 0.23; Thyroid, GLCP = 0.26; Brain_Spinal_cord_cervical_c-1, GLCP = 0.34) and *GPNMB* (Pituitary, GLCP = 0.24; Cells_Cultured_fibroblasts, GLCP = 0.22) showed suggestive, tissue-restricted colocalization signals, consistent with their moderate posterior probabilities from fine-mapping. Although the GLCPs did not exceed 0.5, both genes demonstrated functional relevance in TWAS and/or GWAS annotations, suggesting that the lack of stronger colocalization likely reflects limited power rather than absence of regulatory relevance, and may indicate possible tissue-specific regulatory effects in non-anxiety TMD.

### Proteome-wide association analysis identifies protein-level mediators of TMD genetic components

To identify protein-level mediators underlying the F_Anxiety_ and F_Non-Anxiety_ genetic components, we examined PWAS results obtained using the BLISS framework.

**Fig. 4:**
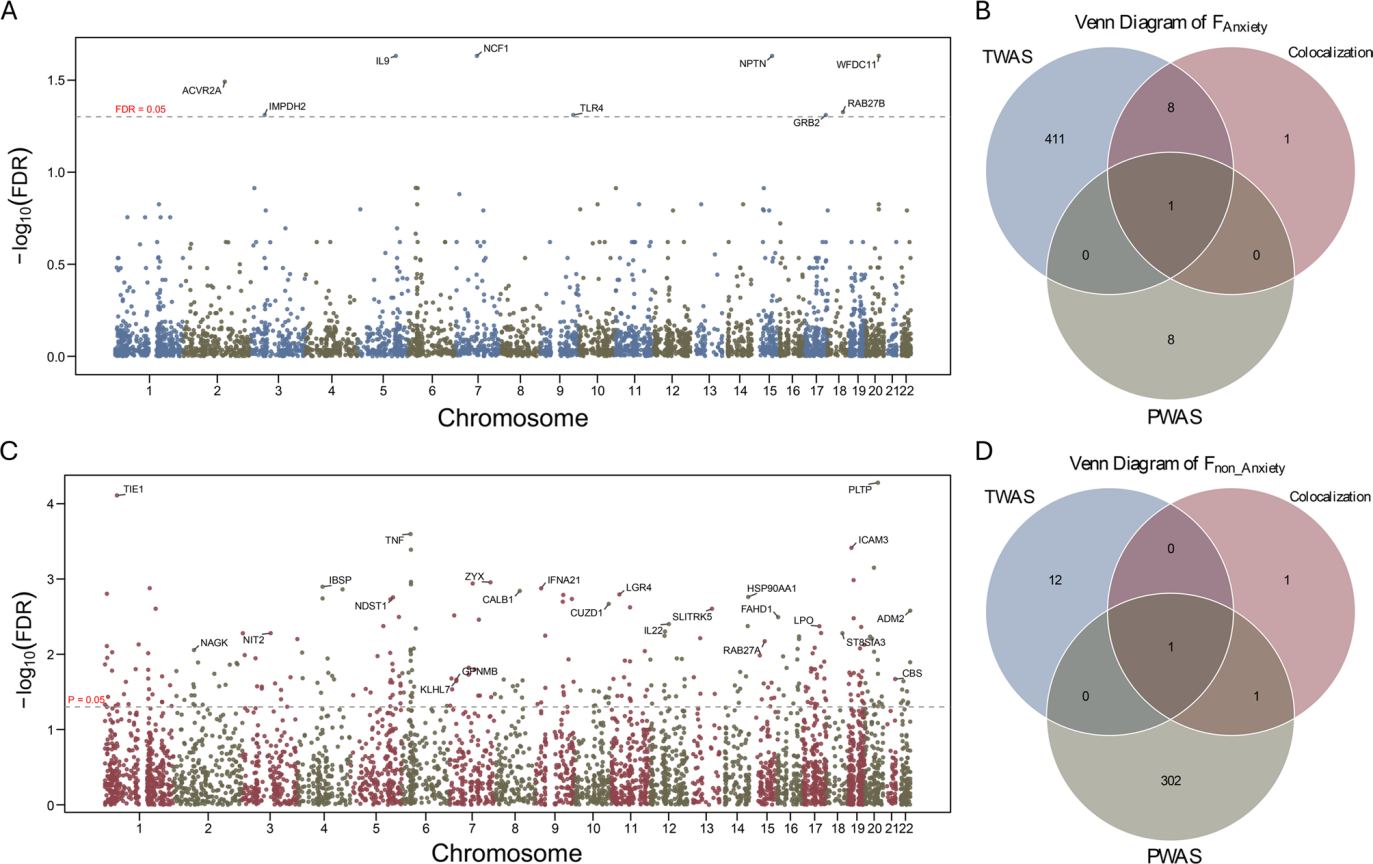
Proteome-wide association results and multi-omics Integration/Cross-validation of anxiety-dependent and anxiety-independent genetic components of TMD. (**A**): Proteome-wide association study (PWAS) results for anxiety-dependent TMD. TMD. TMD. TMD. The red dashed line marks the significance threshold at FDR = 0.05. Genes labeled above this threshold represent significant associations. (**B**): The venn diagram shows the overlap among candidate genes identified by TWAS, PWAS, and colocalization analysis. Only one gene (*RAB27B*) overlapped across all three methods. (**C**): Proteome-wide association study (PWAS) results for anxiety-independent TMD. The red dashed line marks the significance threshold at *p* = 0.05. Genes labeled above this threshold represent significant associations. A total of 304 proteins show nominal-level associations (*p* < 0.05). (**D**): The venn diagram shows the overlap among candidate genes identified by TWAS, PWAS, and colocalization analyzes. Two suggestive genes (*KLHL7* and *GPNMB [in GWAS]*) overlapped across all methods

After multiple-testing correction (FDR < 0.05), nine proteins (*ACVR2A*, *IMPDH2, IL9, NCF1, TLR4, NPTN, GRB2*, *RAB27B*, and *WFDC11*) showed FDR-significant associations with F_Anxiety_ (Fig. 4A, Supplementary Table S5). Among them, the small GTPase *RAB27B* (*p* = 6.40 × 10^−5^, FDR = 0.0471, z = −3.9974) a key regulator of vesicle docking and neurotransmitter release re-emerged as a convergent signal across GWAS, TWAS, and colocalization analyses (Fig0.4 B). This multi-layer convergence supports a potential role for *RAB27B* in anxiety-related genetic liability to TMD.

Other proteins such as *NPTN* (*p* =1.94×10^−5^, FDR = 0.0233, z = 4.2717), *NCF1* (*p* = 8.31×10^−6^, FDR = 0.0233, z = 4.4570), and *WFDC11* (*p* = 2.11×10^−5^, FDR = 0.0233. z = −4.2524) also showed FDR-significant associations (Supplementary Table S5).

In contrast, no proteins reached FDR-significance in the F_Non-Anxiety_ group under the FDR < 0.05 threshold (Fig. 4C, Supplementary Table S5). However, 304 proteins showed nominal significance (*p* < 0.05), including *KLHL7* (*p* = 0.023, FDR = 0.661, z = 2.2789) and *GPNMB* (*p* = 0.022, FDR = 0.661, z = 2.2941). Both proteins were consistently implicated across multiple omics layers, including genome-wide annotation (GPNMB only), transcriptome-wide association analysis (KLHL7 only), and colocalization analysis (both genes) (Fig. 4D, Supplementary Table S5), suggesting their potential involvement in the anxiety-independent genetic architecture of TMD.

### BrainXcan reveals divergent neurostructural associations of TMD genetic components

To characterize the neurostructural correlates of distinct genetic components of TMD, we applied the BrainXcan framework to assess associations between each latent genetic loading and imaging-derived phenotypes (IDPs).

**Fig. 5:**
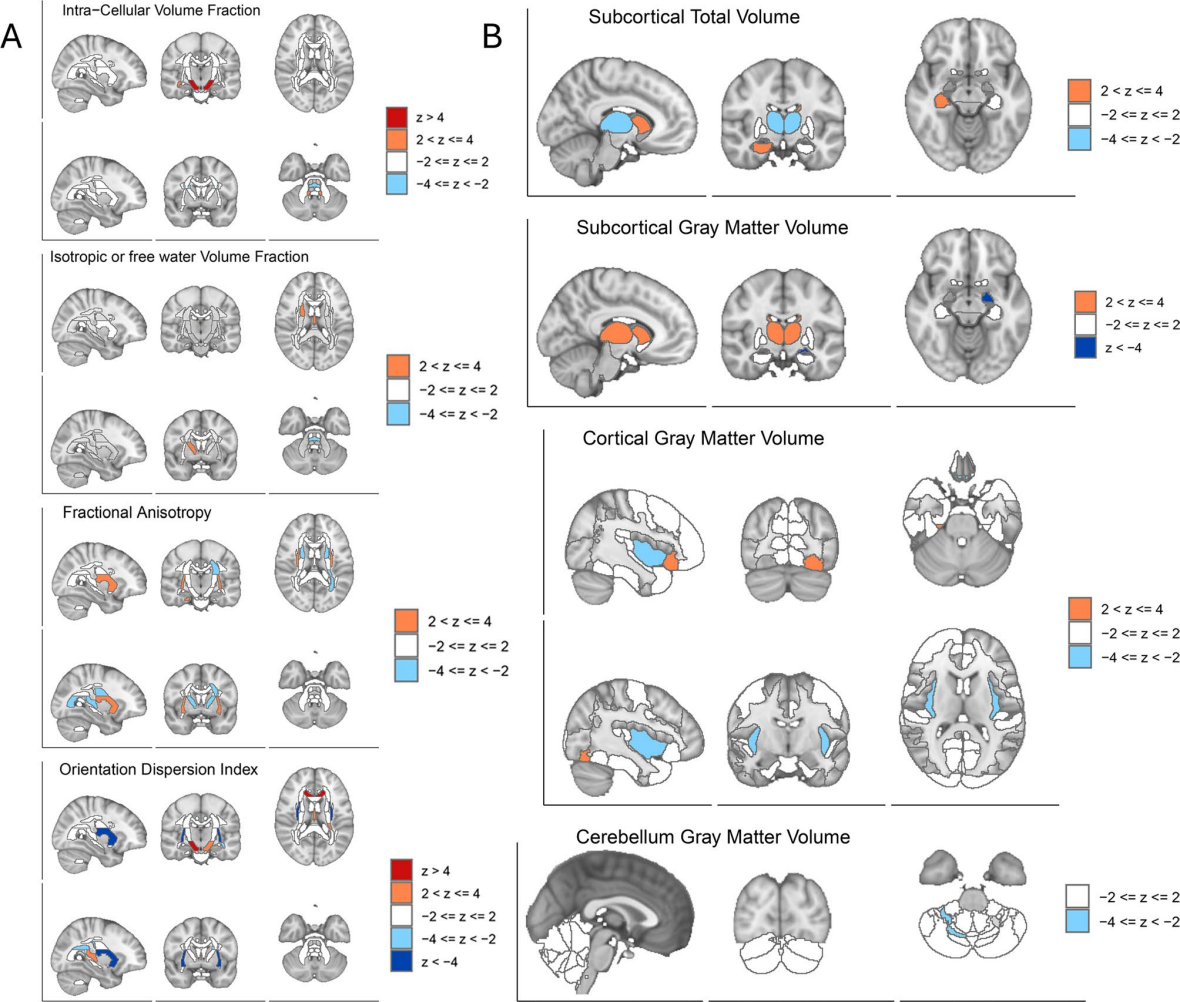
BrainXcan analysis results of anxiety component. (**A**): Associations between F_Anxiety_ (loading 1) and diffusion MRI phenotypes. Orange regions indicate volume increases (2 < z ≤ 4), while blue regions indicate volume decreases (−4 < z ≤–2). (**B**): Associations between F_Anxiety (_loading 1) and T1 structural MRI phenotypes

For F_Anxiety_, a total of 56 IDPs reached FDR significance. Representative associations involved both subcortical gray matter volumes and white matter microstructural measures (Fig. 5 A, B; Supplementary Table S6).The orientation dispersion (OD) of the right external capsule showed a significant negative association with F_Anxiety_ (IDP-25424, *p* = 1.78 × 10^−9^, FDR = 9.70 × 10^−9^, Z = −5.74), as did the left external capsule OD (IDP-25425,*p* = 4.54 × 10^−6^, FDR = 1.32 × 10^−5^, Z = −4.37). Intracellular volume fraction (ICVF) in the cerebral peduncle was positively associated on both the left (IDP-25359, *p* = 1.44 × 10^−5^, FDR = 3.56 × 10^−5^, Z = 4.13) and right sides (IDP-25358, *p* = 1.79 × 10^−5^, FDR = 4.2 × 10^−5^, Z = 4.10). The gray matter volume of the left amygdala exhibited a significant negative association (IDP-25888, *p* = 2.09 × 10^−6^, FDR = 7.03 × 10^−6^, Z = −5.74).

**Fig. 6:**
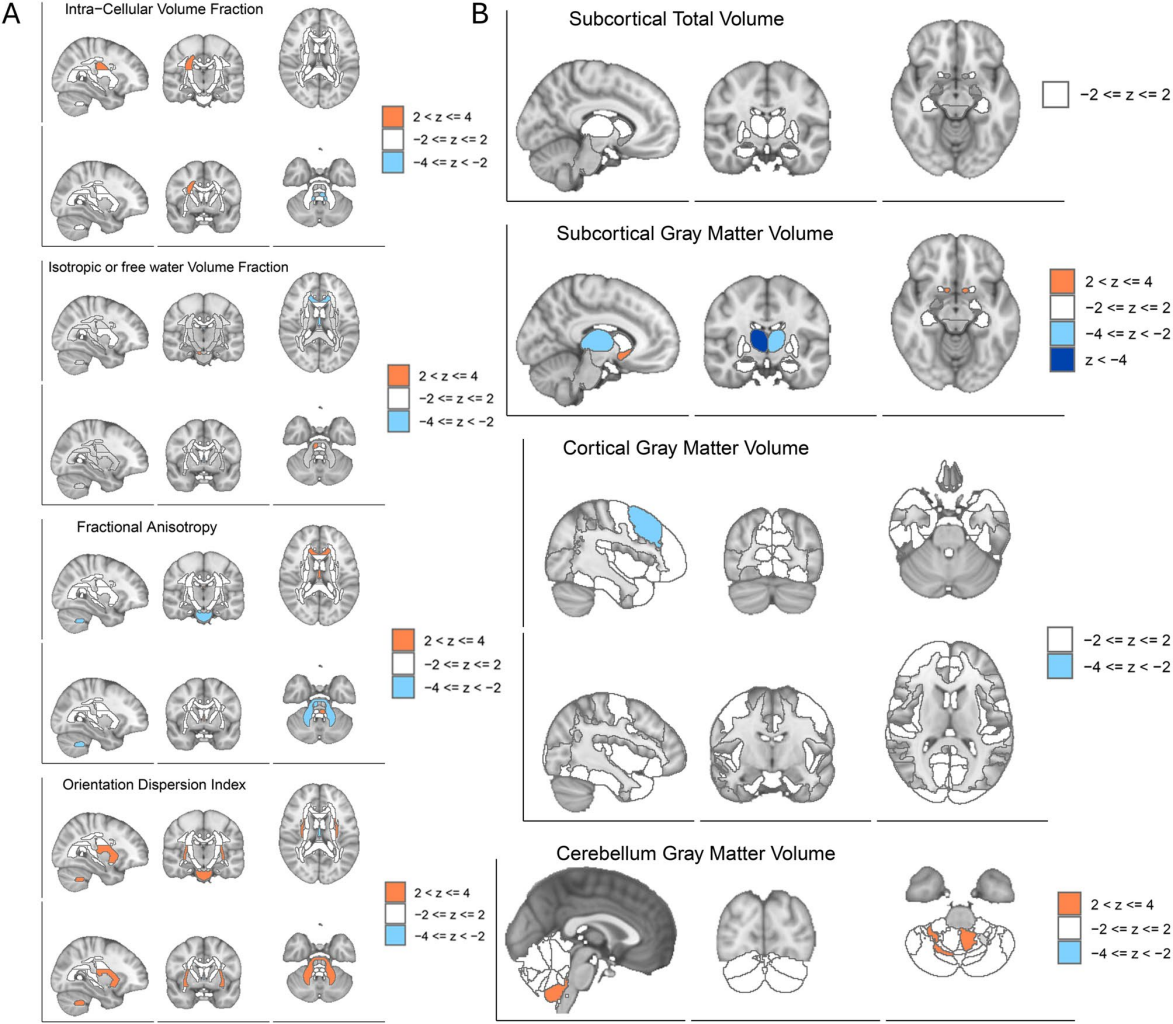
BrainXcan analysis results of non-anxiety component. (**A**): Associations between fnon-anxiety (loading 2) and diffusion MRI phenotypes. Orange regions indicate volume increases (2 < z ≤ 4), while blue regions indicate volume decreases (−4 < z ≤–2). (**B**): Associations between fnon-anxiety (loading 2) and T1 structural MRI phenotypes

In the F_Non-Anxiety_ analysis (Fig. 6A,B, Supplementary Table S6), 41 IDPs reached significance at FDR < 0.05. These genetic components are predominantly within cerebellar gray matter and select subcortical nuclei. Right thalamic gray matter volume showed the negative association (IDP-25879, *p* = 2.24×10^−5^, FDR = 5.95×10^−5^, Z = −4.01). Several white matter tracts displayed significant reductions in fractional anisotropy (FA), including the right inferior fronto-occipital fasciculus (IDP-25501, *p* = 4.36×10^−4^, FDR = 8.89×10^−4^, Z = −3.32), corticospinal tract (IDP-25062, *p* = 2.53×10^−3^, FDR = 4.18×10^−3^, Z = −2.86), and corpus callosum genu (IDP-associations were observed in the fornix (IDP-25397, *p* = 2.00×10^−3^, FDR = 3.38×10^−3^, Z = −2.93) and middle frontal gyrus gray matter (IDP-25789, *p* = 2.47×10^−3^, FDR = 3.92×10^−3^, Z = −2.88). Conversely, the right parahippocampal part of the cingulum exhibited a positive association (IDP-25495, *p* = 2.97×10^−3^, FDR = 4.84×10^−3^, Z = 2.82), suggestive of compensatory remodeling0.25442, *p* = 4.49×10^−4^, FDR = 9.06×10^−4^, Z = −3.32). Concurrent negative

Collectively, F_Anxiety_ associations predominantly involve subcortical and limbic structures, whereas F_Non-Anxiety_ associations are concentrated in thalamo–frontal–white matter networks, highlighting dual-track differentiation of TMD genetic effects on brain structure.

### Cell-type expression mapping provides developmental context for TMD genetic pathways

**Fig. 7:**
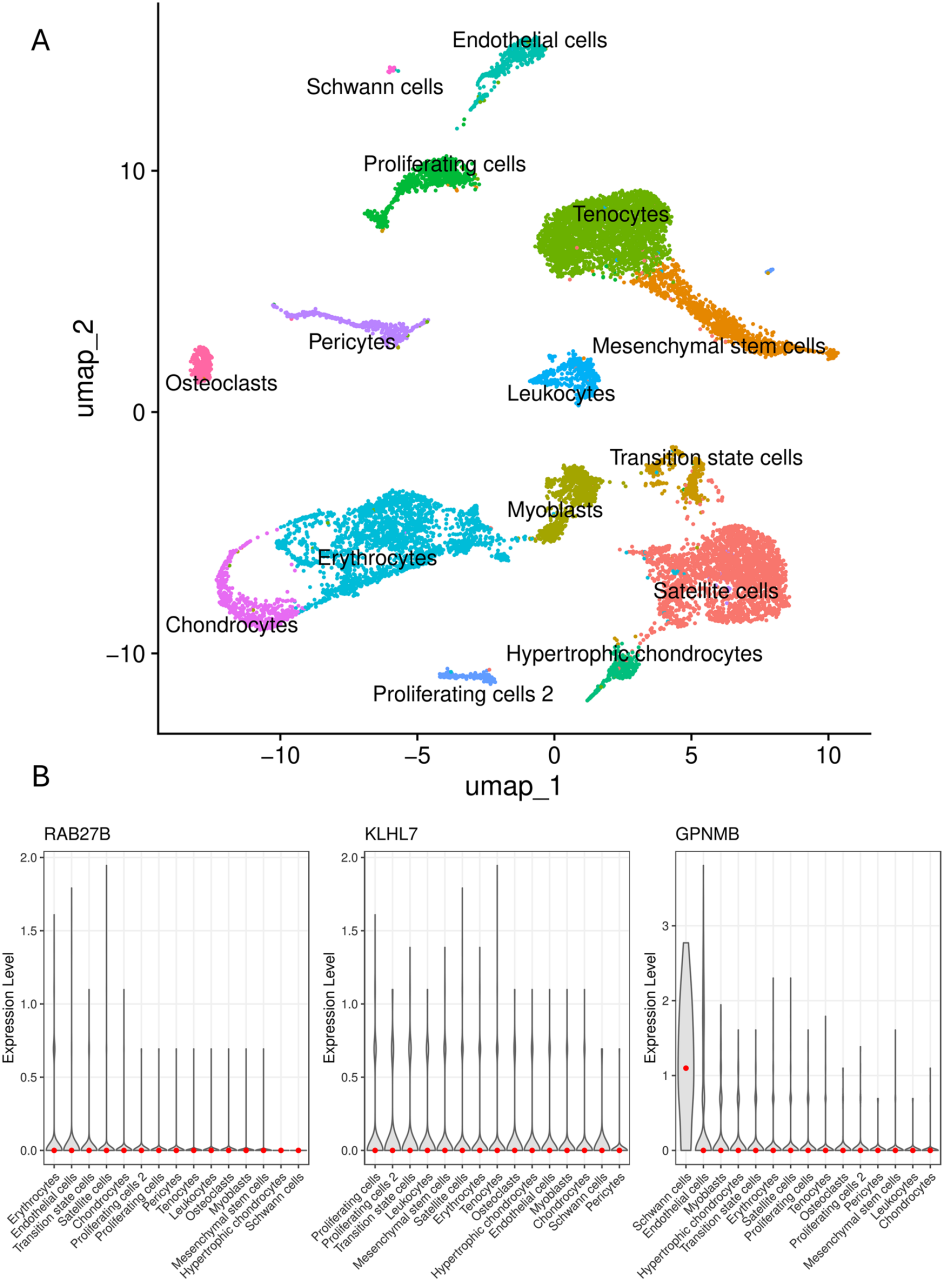
Single-cell expression landscape of prioritized genes in fetal craniofacial and brain tissues. (**A**) UMAP-based clustering of scRNA-seq data reveals distinct transcriptional populations, including schwann cells, tenocytes, mesenchymal stem cells, satellite cells, chondrocytes, hypertrophic chondrocytes, osteoclasts, endothelial cells, pericytes, leukocytes, myoblasts, transition state cells, erythrocytes, and two proliferative cell clusters. (**B**) Cell-type–specific expression patterns of prioritized genes are shown across the atlas. *RAB27B* is enriched in erythrocytes, satellite cells, transition state cells, and endothelial cells. *KLHL7* displays broad expression across multiple lineages, with notable enrichment in tenocytes, satellite cells, myoblasts, transition state cells, endothelial cells, mesenchymal stem cells, leukocytes, and proliferative populations. In contrast, *GPNMB* shows a more restricted pattern with high expression in schwann cells, suggesting distinct cell-type–specific roles in the genetic architecture of temporomandibular disorders

To further contextualize the key genes underlying the F_Anxiety_ and F_Non-Anxiety_ genetic components, we mapped their expression patterns across the 15 cell types identified in the human embryonic TMJC single-cell atlas. This data set comprises 16,624 cells from 3 and 4 month old human embryos, delineating major populations including satellite cells, mesenchymal stem cells, transition state cells, myoblasts, chondrocytes, hypertrophic chondrocytes, tenocytes, endothelial cells, Schwann cells, erythrocytes, leukocytes, osteoclasts, and proliferative subclusters (Fig. 7A). We utilize this embryonic atlas as a representative framework for developmental programming and early tissue biology, acknowledging that while it provides high-resolution cellular insights, it may not serve as a direct proxy for the mature homeostatic or pathological states of adult TMJ tissues.

*RAB27B* (Fig. 7B) displayed a distinct expression profile, with highest abundance in satellite cells, and additional enrichment in erythrocytes, transition state cells, and endothelial cells, suggesting roles in early myogenic or vascular-associated processes.

*KLHL7* (Fig. 7B) showed moderate-to-high expression across multiple lineages, including tenocytes, erythrocytes, satellite cells, myoblasts, transition state cells, endothelial cells, MSCs, leukocytes, and proliferating cells. Notably, tenocytes and satellite cells exhibited the strongest expression peaks, indicating involvement in tendon–muscle developmental interfaces.

*GPNMB* (Fig. 7B) demonstrated a highly cell-type-specific pattern, with robust expression in endothelial cells and Schwann cells, and a striking expression peak in erythrocytes, consistent with functions related to vascular development, neural crest–derived lineages, and early erythroid maturation.

Together, these cell-type–specific expression profiles provide mechanistic context for the distinct anxiety-related and anxiety-independent genetic pathways, highlighting how central neural, vascular, and musculoskeletal cell populations may differentially contribute to TMD heterogeneity.

## Discussion

In this study, we provide first genomic insights into the potential genetic divergence between anxiety-related and anxiety-independent forms of TMD. Leveraging a large-scale genomic data set and a GWAS-by-subtraction framework, our analysis explores a long-standing discussion in orofacial pain biology: whether anxiety in TMD represents a secondary comorbidity or aligns with a distinct biological architecture. By partitioning TMD liability into anxiety-related and anxiety-independent components, we identified that these clinical presentations are associated with distinct genetic signatures, diverging neuroanatomical correlates, and specific cellular expression patterns. These findings offer an alternative to the traditional “unitary” view of TMD pain, suggesting that the disorder may be characterized by at least two potential biological types: a “central-affective” pattern associated with synaptic and limbic system correlates, and a “peripheral-structural” pattern linked to musculoskeletal and sensory relay alterations, consistent with recent conceptual frameworks [9]. Rather than establishing definitive causal pathways, our results provide a biologically informed foundation for generating hypotheses in future mechanistic and prospective clinical studies [9].

### The central-affective component: synaptic plasticity and limbic dysregulation

The anxiety-dependent component F_Anxiety_ exhibited a highly polygenic architecture enriched for genes involved in synaptic transmission and neuroplasticity. A convergent “star” candidate emerging from our multi-omics interrogation is *RAB27B*. This gene, validated across GWAS, TWAS, PWAS, and colocalization analyzes, encodes a GTPase involved in vesicle trafficking and neurotransmitter release. Rab GTPases regulate synaptic vesicle cycling and receptor surface availability in the central nervous system, forming a molecular basis for synaptic plasticity and central sensitization. *RAB27B* cooperates with Rab3 to mediate calcium-dependent neurotransmitter release, and Rab proteins require geranylgeranylation for proper membrane localization and activation. Variants that disrupt this modification may impair vesicular transport and alter synaptic transmission efficiency. Through these mechanisms, *RAB27B* can modulate neuronal excitability and facilitate central sensitization, thereby amplifying pain processing [38–40]. The identification of *PCLO* (a presynaptic cytomatrix protein) and *PRSS16* further reinforces the hypothesis that anxiety-associated TMD is driven by synaptic gain-of-function [41–44].

From a clinical perspective, such centrally mediated amplification is expressed as a spectrum of overlapping pain phenotypes in TMD, including arthralgia, disc displacement, and osteoarthritic changes, frequently accompanied by myalgia and myofascial pain. This phenotypic convergence reflects the integrated functional organization of the temporomandibular joint, masticatory muscles, tendons, and ligamentous structures, indicating that TMD-related pain arises from coordinated dysfunction across multiple tissues rather than from isolated joint pathology. These features provide a clinical rationale for extending mechanistic investigations beyond TMJ tissue alone and motivate single-cell analyses that encompass the broader stomatognathic system.

Within this multilevel framework, our PWAS results further implicate a neuro-immune interface in anxiety-related TMD. Elevated F_Anxiety_ genetic risk was associated with altered plasma levels of *RAB27B* and inflammatory mediators such as *NCF1* and *TLR4*, consistent with peripheral immune signaling interacting with central neuroplasticity [45, 46]. Furthermore, the enrichment of *RAB27B* in satellite cells of the TMJ condyle suggests that peripheral glial–neuronal crosstalk may serve as an initiating node linking local tissue responses to central sensitization.

### Neuroanatomical substrates of anxiety-related TMD

Our BrainXcan analysis provided further structural context to these genetic findings. F_Anxiety_ was significantly associated with reduced gray matter volume in the amygdala and altered microstructure in the external capsule. The amygdala is recognized as the hub of the “emotional brain,” often linked to fear conditioning and the emotional shaping of pain [47–50]. Atrophy or dysfunction in the amygdala is a hallmark of chronic pain states comorbid with anxiety, which may relate to the excitotoxic effects of sustained stress signaling [51, 52]. The specific involvement of the external capsule, a white matter tract connecting the striatum and cerebral cortex, could suggest potential disruption in the connectivity of networks regulating emotional regulation and pain coping [53, 54]. Together, these data indicate that patients with anxiety-related TMD suffer from a “connectopathy” of the limbic system, where the brain’s ability to dampen nociceptive signals is genetically compromised. Together, these data indicate that patients with anxiety-related TMD may share a genetic liability for a limbic “connectopathy,” in which the brain’s capacity to dampen nociceptive signals is potentially compromised. Because BrainXcan leverages genetically driven variation rather than disease-induced changes, these associations are more consistent with predisposing neuroanatomical correlates rather than mere consequences of chronic pain. However, we emphasize that these BrainXcan associations are not necessarily causal and may reflect horizontal pleiotropy or a shared genetic liability between brain morphology and TMD risk, rather than a direct mechanistic pathway.

### The anxiety-independent component: peripheral pathology and structural integrity

In striking contrast, the anxiety-independent component F_Non-anxiety_ revealed a distinct, less polygenic profile rooted in structural biology. The most prominent genetic signals were associated with *GPNMB* (Osteoactivin) and *KLHL7*. *GPNMB* is a transmembrane glycoprotein critical for osteoblast differentiation, bone mineralization, and tissue repair [55]. The association of this candidate with non-anxiety TMD suggests that in this subgroup, pain may be linked to processes such as bone remodeling or localized inflammatory responses at the joint level, potentially independent of central psychological modulation. Similarly, *KLHL7*, a component of the ubiquitin-proteasome system, implies a role in protein homeostasis essential for maintaining musculoskeletal tissue integrity under mechanical stress [56]. Given these functions, it is plausible that both genes are involved in pathways associated with responses to parafunctional loading—such as bruxism—or to muscle-driven mechanical activation at the periosteal–bone interface. Such mechanisms offer a potential biological framework for understanding how repetitive masticatory muscle activity and excessive mechanical strain might correlate with peripheral tissue changes observed in anxiety-independent TMD. The cellular mapping of these genes further aligns with this “peripheral” hypothesis. *KLHL7* exhibited peak expression in tenocytes and satellite cells, while *GPNMB* showed high expression in erythrocytes and endothelial cells within the TMJ condyle. This localization suggests a potential genetic vulnerability at the tendon-muscle interface and vascular microenvironment. Clinically, these findings suggest that TMD unaccompanied by anxiety may be characterized by biomechanical failure and localized tissue inflammation, rather than being primarily driven by central amplification. This perspective aligns with clinical observations in patients exhibiting bruxism or other parafunctional loading behaviors, where repetitive masticatory muscle activity may impose excessive mechanical stress on the TMJ and surrounding tissues, thereby correlating with the peripheral signature’s characteristic of anxiety-independent TMD.

### Divergent brain signatures in non-anxiety TMD

The neuroimaging associations for F_Non-anxiety_ were notably distinct from the limbic patterns observed in the anxiety group. This component exhibited a significant association with structural alterations in the thalamus (the primary sensory relay center) and the corticospinal tract. Given that the thalamus acts as a gateway for nociceptive input to the cortex, these alterations are consistent with a potential dysregulation in the raw transmission of sensory data, rather than its emotional processing [18, 57–59]. Furthermore, the involvement of motor pathways (corticospinal tract) and cerebellar gray matter suggests that anxiety-independent TMD may share a genetic liability for maladaptive motor control of the jaw. This potentially links the condition to parafunctional habits, such as bruxism, which are mechanical rather than purely affective in origin. Genetically driven variances in dopaminergic (e.g., *DRD2*) or serotonergic signaling (e.g., *HTR2A*), both of which have been implicated in bruxism [60, 61], may predispose individuals to such disinhibition within the thalamo-motor loops [62, 63], These genetic factors may thus provide a molecular scaffold for the observed structural plasticity in motor tracts, appearing independent of limbic interference. As with the anxiety-related findings, these BrainXcan associations should be interpreted as genetically driven correlates rather than definitive causal pathways, acknowledging the potential for horizontal pleiotropy.

### Clinical implications: toward precision medicine

Current management of TMD often employs a “trial-and-error” approach, treating all patients with similar protocols of Supported Self Management [64], splint therapy, NSAIDs, or physical therapy [65]. Consideration of Axis II factors, namely psychosocial distress, has been established as a standard component of comprehensive care for patients with TMD-related pain [66]. Specifically, the F_Anxiety_ component identifies a central-affective type driven by synaptic and neuroimmune pathways, suggesting that for TMD patients with high anxiety load, future clinical strategies could extend beyond symptom-based counseling. Clinicians might consider early screening for these “central” genetic predispositions. For this subgroup, management could be tailored toward early-stage neuromodulatory treatments, such as antidepressants or anticonvulsants with analgesic properties, or targeted cognitive-behavioral interventions aimed at modulating limbic–synaptic circuits. In contrast, the F_Non-anxiety_ component highlights musculoskeletal remodeling and peripheral inflammatory mechanisms. Patients predominantly exhibiting this genetic signature may benefit more from restorative biomechanical stabilization, including precision splint therapy to reduce joint loading, along with localized anti-inflammatory interventions or physical therapies targeting temporomandibular joint tissue remodeling. While these pathways suggest a potential roadmap for precision TMD care, they remain hypothesis-generating frameworks that require rigorous prospective validation before adoption as clinical protocols.

### Strengths and limitations

A key strength of this study is the application of GWAS-by-subtraction to disentangle correlated genetic liabilities, enabling separation of anxiety-related and anxiety-independent components within TMD at the population level. This framework is further strengthened by an integrative multi-omics approach linking genetic variation to transcriptional (TWAS), proteomic (PWAS), neurostructural (BrainXcan), and cell-type–specific (scRNA-seq) contexts, providing convergent evidence across multiple biological layers.

Several limitations warrant consideration. First, the FinnGen TMD definition (ICD-10 K07.6) captures clinically heterogeneous presentations, including both painful and non-painful cases. Because individual-level symptom data are unavailable, we were unable to stratify cases by pain status or directly quantify pain-related genetic effects. Second, anxiety was modeled as a broad, cross-disorder phenotype; thus, the FAnxiety component likely reflects a shared genetic core across anxiety-related conditions, with possible contribution from depressive traits.

Third, although the underlying FinnGen GWAS uses a standard case–control design with non-TMD controls, the present analyses do not constitute a new case–control comparison. Instead, GWAS-by-subtraction partitions genetic liability within TMD into orthogonal latent components. Accordingly, the findings should be interpreted as associative genetic stratification rather than causal effects or clinically observable subtypes. Establishing causality or clinical specificity will require future studies with individual-level data and detailed phenotyping.

Additional limitations include reliance on summary-level GWAS data, which precludes direct modeling of individual-level heterogeneity, and the interpretive constraints of statistical colocalization, which indicates shared variants rather than biological causality. Finally, single-cell analyses were restricted to embryonic TMJ complex tissues due to the lack of adult human TMJ scRNA-seq data; these results are therefore interpreted as reflecting developmental programming and lineage context rather than adult disease states.

### Conclusion

In conclusion, our findings suggest that TMD may not be a unitary genetic entity but rather a heterogeneous condition associated with at least two distinct biological signatures. The presence of anxiety in TMD may be viewed not merely as a comorbid symptom, but as a potential marker of a specific genetic architecture characterized by synaptic and limbic correlates. This architecture is distinct from the musculoskeletal and sensory-relay liabilities observed in anxiety-independent TMD. Rather than establishing definitive causal pathways, these findings provide a biologically informed framework for understanding the heterogeneity of TMD. This framework may offer a foundation for future precision medicine approaches that consider the diverse genetic liabilities underlying an individual patient’s pain profile.

## Electronic supplementary material

Below is the link to the electronic supplementary material.

Supplementary material 1

## Data Availability

The summary-level GWAS data sets analyzed in this study are publicly available. TMD GWAS data were obtained from the FinnGen consortium (Release 12, https://www.finngen.fi/en). GWAS summary statistics for anxiety were obtained from a previously published study (10.6084/m9.figshare.23659653.v1). All summary statistics generated in this study, along with the complete fine-mapping and colocalization results, are available in a publicly accessible repository (DOI: 10.5281/zenodo.18765836). Variant annotation resources included dbSNP build 157 (https://ncbiinsights.ncbi.nlm.nih.gov/2025/03/18/dbsnp-release-157/) and the 1000 Genomes Project Phase 3 reference panel (https://www.internationalgenome.org/category/phase-3/). Transcriptome-wide association analyzes were performed using GTEx v8 reference transcriptomic data and pre-trained FUSION expression weights (http://gusevlab.org/projects/fusion/). Colocalization analyzes were conducted using fastENLOC (https://github.com/xqwen/fastenloc). Single-cell transcriptomic data were obtained from the Gene Expression Omnibus under accession number GSE308079. Proteome-wide association analyzes (PWAS) used BLISS models publicly released by Chongwu Lab (https://huggingface.co/data sets/chongwulab/BLISS-models).

## Abbreviations

GWAS: Genome-wide association study
GSUB: GWAS-by-subtraction
SuSiE: Sum of single effects
TWAS: Transcriptome-wide association study
PWAS: Proteome-wide association study
BLISS: Biomarker expression level imputation using summary statistics
scRNA-seq: Single-cell RNA sequencing
TMD: Temporomandibular disorders
FAnxiety: Anxiety-related genetic component
FNon-Anxiety: Anxiety-independent genetic component
IDPs: Imaging-derived phenotypes
eQTL: Expression quantitative trait locus
GLCP: Gene–locus colocalization probability
LD: Linkage disequilibrium
MAF: Minor allele frequencydb
SNP: Single Nucleotide Polymorphism Database
ICD-10: International Classification of Diseases, 10th Revision
DSM: Diagnostic and Statistical Manual of Mental Disorders
GAD-2: Generalized Anxiety Disorder-2
DC/TMD: Diagnostic Criteria for Temporomandibular Disorders
ICOP: International Classification of Orofacial Pain
MRI: Magnetic resonance imaging
GTEx: Genotype-Tissue Expression Project
UKB-PPP: UK Biobank Plasma Proteome Project
UMAP: Uniform manifold approximation and projection
FDR: False discovery rate
ICVF: Intracellular volume fraction
OD: Orientation dispersion
FA: Fractional anisotropy
TMJC: Temporomandibular joint condyle

## Code availability

All analyses were conducted using established, publicly available software packages, including tools for GWAS-by-subtraction, TWAS, PWAS, colocalization, BrainXcan analysis, and single-cell RNA-seq processing. No new software or computational methods were developed in this study. The analyses followed standard implementations and recommended workflows provided by the original software developers, which are available through the respective project websites and repositories. Custom code used in this study is available upon reasonable request.

## Declarations

### Declaration of AI assistance

During the preparation of this work, the authors used AI-assisted tools (ChatGPT, OpenAI) solely for language editing and text refinement in the preparation of the cover letter and certain non-scientific textual elements. After using this tool, the authors carefully reviewed and edited the content as needed and take full responsibility for the content of the published article.
